# Prediction as Internal Simulation: Taking Chances in What to Do Next

**DOI:** 10.3389/fpsyg.2012.00405

**Published:** 2012-10-18

**Authors:** Malte Schilling, Katharina Rohlfing, Holk Cruse

**Affiliations:** ^1^Center of Excellence “Cognitive Interaction Technology,” Bielefeld UniversityBielefeld, Germany

What’s next? – To know what comes next is already important in carrying out action and allows us to make fast movements. We use predictions in control of our own movements and to anticipate what is going on around us.

Clark’s (in press) perspective is pushing the importance of prediction even further. Predictions are the “language” in between different representations: levels of representation do not communicate by exchanging state information, instead the information flow is lazy and one level is telling the other only what that does not know – or would not predict or expect.

While we value Clark’s thoughts on the hierarchical prediction machine approach and its implications, one important requirement is not addressed: the connections between different levels of representations are not only not arbitrary, but it appears that these can be flexibly utilized and in this way different combinations of interconnected – predicting – layers can serve diverse purposes.

To illustrate, we put this issue into the viewpoint of representations, central to which functional internal models are. We agree with Clark that these internal models have to be predictive. But there may be more to them then only prediction. First of all, engaged (and maybe grounded as sensorimotor circuits) internal models may serve an inverse function in motor control, i.e., coming up with motor control commands when given a certain goal. Secondly, as animals and humans have a wide variety of redundant sensors, such internal models should exploit the redundancy and integrate the noisy contributions of multiple sensors.

As we know today, such internal models are not only serving one single function, but internal models are recruited in service for diverse function (Anderson, 2010), e.g., perception, to understand the actions performed by somebody else, or in planning ahead. The internal models are recruited in an internal simulation (Hesslow, 2002) – and central is their predictive function: in planning ahead they are used to simulate possible consequences of actions and then to choose only a suitable one. In understanding someone’s else actions they are driven by an unfolding action and start to resonate with the action, at the same time also invoking representations related to this action, e.g., on a higher level something like a goal or guiding perception on a lower level. While prediction is essential to internal models, it is the flexible use and the way in which levels can be combined and inform each other that makes this a powerful tool serving many functions [up to language, as shown, e.g., by Ramscar et al. (2010)]. This flow of predictive information in between levels (top-down and bottom-up) of representation depends on both: on the one hand, on the capability to activate connected levels of representation (e.g., attention), but on the other hand, also to decouple levels of representation (for example, in planning ahead it is important that the body itself is decoupled from the planning process, but we can plan on different levels and switch between these levels). These mechanisms are left out in Clark’s approach and should be specified next. Yet, if we would like to scale a model up to symbolic representations such as language, we need to understand how connected levels of representation are linked and inform each other in multiple ways, and how the connections can be modulated and are flexible in different contexts.

We approach these connections of internal representation through models of minimal cognitive systems in a bottom-up approach (Schilling and Cruse, submitted;Schilling and Cruse, 2008). An early neural network model of the own body is grounded in a biological-inspired framework that controls walking in a hexapod robot. While this internal model is very simple, it is quite flexible and can be recruited in service for multiple functions (Schilling, 2011). Importantly, the predictive capabilities of the body model allow applying the model in internal simulation. In internal simulation alternative possible behaviors can be tried out and the predicted consequences can be evaluated without actually carrying out probably dangerous behaviors (Hesslow, 2002). The system can plan ahead and becomes cognitive in the sense of McFarland and Bösser (1993). Interestingly, the behavioral system, which allows variation of existing or creation of new behaviors, implements a form of unified neuronal workspace (Dehaene and Naccache, 2001; on a more abstract level this might be termed a global workspace following; Baars and Franklin, 2007). In this global workspace, behavioral elements can be accessed (and possibly varied) in new contexts in order to find a (new) solution to an actual problem. The decisions of the system are not directly triggered by sensory input and are not predictable from outside. Instead action selection occurs at various levels of complexity. We argue that such a system shows properties of what has been termed access consciousness by Cleeremans (2005; which is distinguished from metacognition and phenomenological consciousness). Access consciousness refers to the ability of a system to plan and guide actions, to reason and to report verbally on the content of the corresponding representations. Such accessible states are required to guide the action variation process during internal simulation. They emerge in such a system leading to networks of interconnected higher level representations which are at the same time grounded, but not directly accessible, lower level sensorimotor representations. Competitive activation of representational units leads to an attention-like focus. From our point of view, it is interesting how such simple neural network based models show such high-level properties and connect to philosophical accounts^1^. Even though internal models are central to this approach, it is their flexibility which makes the system cognitive. Internal simulation becomes crucial, as the decoupled use of internal models in internal simulation suddenly allows evaluating alternative actions. This introduces an internal competition of alternative behaviors and varying existing behaviors or coming up with new behaviors (even risky ones) becomes now valuable. The concurrent activation of such behaviors leads, first, to action selection. And second, a form of attention as a focus on this behavior emerges and the behavior has to be evaluated. The unfolding behavior has to be related to the agent’s motivations or, on a higher level, goals. Prediction is the key to, first, allow for planning ahead as internal simulation and choosing novel behaviors only based on estimated consequences. Second, this might serve as a starting point for a higher level organization of accessible and attended internal states.

## Footnotes

^1^Which currently guides future research as we are specifically addressing two topics: first, connecting high level (“consciously aware”) states and use information in a simple communication which allows the system to utter its current state or through which the process of internal simulation can be guided externally. Second, with respect to metacognition, internal simulation provides a starting point for a theory of mind.
